# Effective cloth folding trajectories in simulation with only two parameters

**DOI:** 10.3389/fnbot.2022.989702

**Published:** 2022-09-08

**Authors:** Victor-Louis De Gusseme, Francis wyffels

**Affiliations:** IDLab-AIRO, Department of Electronics and Information Systems, Ghent University – imec, Ghent, Belgium

**Keywords:** cloth, folding, simulation, robotic, manipulation, trajectory

## Abstract

Robotic cloth folding remains challenging for robots due to its highly deformable nature. In order to deal with these deformations, several strategies with varying amounts of adaptability have been proposed. We study robotic cloth folding by simulating and evaluating a trajectory search space with only two parameters: one parameter for the trajectory's height and one parameter to tilt it. We extensively analyzed folding a long-sleeved shirt in a high-fidelity simulator. To demonstrate that the trajectory is sufficiently adaptable and robust, we test several cloth shapes, cloth materials, an entire folding sequence and different folding velocities. We can deal with every folding scenario by tuning the two parameters correctly. The trajectories' simplicity and their robustness in simulation make them ideal candidates for future transfer to real-world robotic setups.

## 1. Introduction

Handling cloth with robots is still challenging because cloth behavior is hard to predict. This unpredictability is due to cloth's high deformability and the numerous self-contacts it encounters when being manipulated. Various complications arise when folding cloth: parts of it can slide and shift, it can crumple, wrinkles can appear and it can buckle in unexpected ways (Jiménez, 2017). When multiple layers of fabric are stacked on top of each other, these challenges become even more pronounced. Cloth manipulation has many characteristic failure modes that affect rigid objects or even other deformables to a much lesser extent. Therefore, manipulation strategies for these more standard materials cannot be applied to cloth directly.

In this work, we seek to advance robotic cloth folding. We do this by carrying out an in-depth analysis of the folding performance of a simple trajectory search space. This analysis is performed entirely in simulation. Simulation provides a controlled, informative, and reproducible environment for our experiments (Narain et al., 2012; Matas et al., 2018; Antonova et al., 2021; Lin et al., 2021; Seita et al., 2021). This is especially important for cloth, because its state is notoriously hard to estimate in the real world exact experiments are very hard to reproduce. Our results show that for successful cloth folding in simulation, it is essential to adapt to the cloth's shape, material and to the specific folding step within a sequence.

Several prior works have investigated robotic cloth manipulation. Maitin-Shepard et al. (2010) and Doumanoglou et al. (2016) have described entire robotic cloth folding pipelines that go from piles of crumpled clothes to nicely folded stacks. Naturally, one of the essential subtasks in these folding pipelines is the folding of the flattened garments. Both pipelines use static and heuristic trajectories for the folding motion. These heuristics are fine for towels but can still be improved for more complex garments. Folding as robustly as humans requires a remarkable amount of subtility and adaptability.

Others have proposed several heuristic strategies and trajectories for robotic cloth folding (Berg et al., 2010; Petrík et al., 2015). For example, Berg et al. (2010) described a simple procedure named gravity-folds. It depends on gravity to keep parts of the cloth in a predictable vertical position during folding. However, such procedures based on geometric reasoning about cloth have their limitations: cloth often violates the assumptions about its material properties that these methods require to succeed. This problem is acerbated when the folding motion has any appreciable speed, which makes the dynamic behavior of cloth emerge. Petrík and Kyrki (2019) have shown that adaptability to the specific material properties of cloth is crucial for folding success and that we cannot afford to abstract away cloth physics. More recently, several authors have applied reinforcement learning to the problem of cloth manipulation (Matas et al., 2018; Jangir et al., 2020). These methods propose a single framework capable of learning a range of different tasks. While the generality of these methods is attractive, the learned strategies are often unexplainable, hard to reproduce, and possibly fragile to unseen variations (Henderson et al., 2018). Furthermore, finding suitable reward functions to train reinforcement learning agents for cloth folding is also not trivial (Verleysen et al., 2022).

Simulation offers a controlled setting to study and account for cloth material properties and dynamics. The work most similar to ours is by Li et al. (2015), where they use simulation to inform cloth folding trajectories. They use a gradient-based search strategy in simulation to arrive at a single optimal folding path. However, we argue that it is unlikely the optimal folding path in simulation corresponds to the best strategy for real-world folding. The well-known decrease in performance of robotic control strategies when transferring from simulation to the real world is often referred to as the simulation-to-reality gap (Kadian et al., 2020). For simulated manipulation strategies, robustness is of much greater importance than optimality. Therefore, in this work, we do not seek purely to optimize folding trajectories. Rather, our primary aim is to map out the performance of a reasonable search range of trajectories. This wider view gives us more insight into which simulated strategies are more likely to be robust.

**Figure 1:**
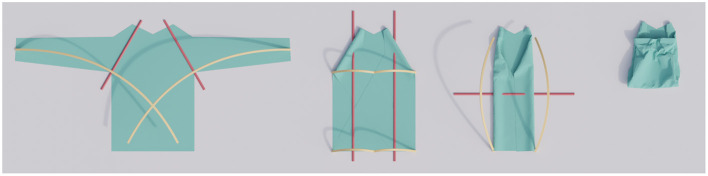
A successful multistep folding sequence for a long-sleeved shirt. The trajectories (yellow curves) are adapted for each step.

In this work, we evaluate a simple folding trajectory with only two parameters based on Bézier curves. We show that this trajectory is sufficiently adaptable to perform well on all our considered variations. Moveover, we show that correctly setting the two parameters is crucial for high performance on all variations of cloth material properties, cloth shapes, and the different folding steps in a sequence. Our method can be applied to arbitrary cloth shapes and fold lines, and at any step in a multistep folding sequence (see Figure 1). In the following, we explain our procedure that goes from the fold specification to the evaluation of the search space. This represents a new methodology for cloth folding in simulation, with a strong focus on future simulation-to-reality transfer. The most important aspect of our work is that we focus on robust folding, as opposed to solely optimal. By reporting folding performances for entire search spaces, we also further the understanding of the problem of cloth folding itself.

To summarize, our main contributions are threefold:

- We are the first to use the C-IPC simulator in the context of robotic manipulation, specifically cloth folding.
- We present an elegant methodology for cloth folding in simulation that includes a novel and effective 2-dimensional trajectory search space.
- We execute an extensive sensitivity analysis of the parameters that influence folding performance, including cloth shape, material, folding phase, and folding speed.

The remainder of this work is structured as follows: in Section 2, we motivate and describe the simulator we chose for this work. In Section 3, we elaborate on our method and our experimental design. In Section 4, we present and discuss the results of these experiments. Finally, in Section 5, we summarize our main findings and their implications for future work and simulation-to-reality transfer.

## 2. Simulation

In this work, we use the high-fidelity codimensional incremental potential contact (C-IPC) simulator (Li et al., 2021), which we believe to be a promising simulator for cloth manipulation research for several reasons. Most importantly, C-IPC handles contact robustly, intersection-free, and accurately even in challenging scenarios. Furthermore, it is based on the finite-element-method (FEM), thus physically grounded, and simulates cloth with realistic finite geometric thickness. To our best knowledge, we are the first to investigate C-IPC for cloth manipulation.

IPC introduced a new method of simulating accurate and interpenetration-free contact for 3D deformables (Li et al., 2020). C-IPC is the extension of the IPC simulator to codimensional, i.e., 1D and 2D, deformables such as cloth and ropes. Simulating these thin materials volumetrically would be less computationally efficient. Additionally it can lead to numerical problems and artifacts, such as shear locking, unless extremely high-resolution meshes are used.

We use C-IPC only for the simulation of cloth. However, C-IPC can simulate the interaction between, ropes, cloth, and volumetric deformables, which could also make it interesting for robotic tasks where these kinds of materials interact. Kim et al. (2022) have already used IPC for grasping rigid objects with soft gripper tips.

C-IPC is an FEM-based method, which means the cloth's internal material model, also called the constitutive model, is a principled discretization of continuum mechanics. This means the simulation method is strongly grounded in reality. An additional benefit of FEM-based simulation is that many simulation parameters correspond to physical parameters such as the material's Young's modulus and Poisson's ratio. This is particularly interesting when trying to match simulation with real-world behavior. However, arguably even more important for cloth folding is not the constitutive model, but how contact and friction are handled. Folding is the act of stacking layers of cloth on top of each other, and thus naturally introduces many contacts. Many folding failure modes can be attributed to contact and friction. For this reason, accurate simulation of those effects is crucial. C-IPC handles both static and kinetic friction, i.e., the friction in both sticking and sliding modes, and rapid switching between these modes.

For C-IPC the cloth needs to be provided as a triangle mesh. For the t-shirts in this work, we use a parametric t-shirt model that generates a polygonal shirt outline from 12 parameters such as sleeve length, sleeve angle, etc. This is similar to the shape models of Miller et al. (2011) and Stria et al. (2014). The interior of this outline is then triangulated with 2D Delaunay triangulation. For all our meshes we run this algorithm with a minimum triangle density of 20K per square meter. For the long-sleeved shirts, this results in meshes with approximately 12K triangles, and for the short-sleeved ones 10K. Higher triangle resolutions produce even finer details, however, we found 20K to give a nice trade-off between simulation time and the smallest possible wrinkle size. A limitation of this work is that garments are simulated with only a single layer of fabric, as opposed to two layers sewn together. We made this choice because simulating garments as several parts sewn together is considerably more complicated and computationally expensive.

The high fidelity of C-IPC makes simulation relatively computationally expensive. We can afford this because of the deliberately limited degrees of freedom of the trajectory and the small size of the search space. We also note that C-IPC is a very recent simulator and could benefit from further optimization and GPU acceleration. One caveat we noticed was that running the simulation with a large ground plane can slow down the simulation by an order of magnitude and affect the result of the simulation. So we recommend using the smallest possible ground plane that fits the simulated cloth.

## 3. Method

**Figure 2:**
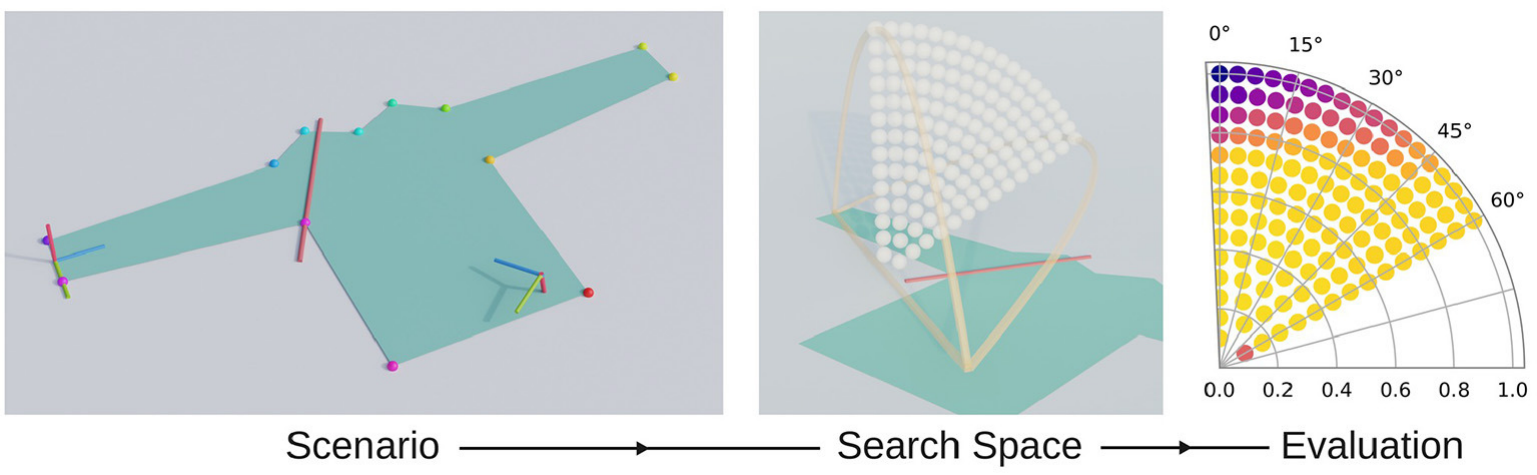
Method overview: a selected scenario **(left)** for which a search space is automatically set up **(middle)** and which is subsequently evaluated **(right)**.

The core of our method is the simulation of fold trajectories and the evaluation of the cloth's state at the end of the simulation. Figure 2 provides a schematic overview of this process. We vary the simulated scenario and look at the effect on trajectory performance. A scenario includes the shape of the simulated piece of cloth, its material parameters, and a fold specification.

A fold specification represents the desired fold to be performed and consists of a fold line and gripper start pose. The fold line and gripper start pose are specified relative to a set of semantic keypoints on the piece of cloth. For a t-shirt, these keypoints could be, e.g., the left armpit, the right shoulder, and other meaningful locations on the shirt. The fold specification is further detailed in Section 3.1.

From the fold specification, we construct a search space of trajectories with only two parameters. One parameter controls the height of the trajectory and one determines by how much to tilt it. For each simulation scenario, we simulate and evaluate a set of 134 trajectories in this search space. This will be the subject of Section 3.2.

For the evaluation of a fold, we compare the resulting simulated mesh to a target mesh. This target represents an idealized folded state where the part to be folded is geometrically rotated by 180 degrees. To compare the result and the target we calculated the mean distance between corresponding vertices. We elaborate on the evaluation in Section 3.3.

We end the section with the experiment setup in Section 3.4. There we explain our reasoning for the 13 evaluated scenario variations.

Algorithm 1 shows the procedure of our method for a single scenario.

**Algorithm 1:** Evaluation procedure for a single scenario

**Input**: Scenario and fold specification
**Output**: Folding performance of the trajectories in the search space
**1** Design a scenario (including cloth shape and material properties)
**2** Select a fold line and gripper start pose
**3** Calculate the target mesh for the fold line
**4** Construct the search space
**5** Simulate the effect of each trajectory
**6** Compare the simulated results with the target mesh

### 3.1. Fold specification

In this work, we study the motions required to accomplish a predefined fold. Which fold or folding sequence is desirable is subject to personal preference. We encode this information as a fold specification, similar to Petrík et al. (2017). It consists of a fold line and a gripper start pose (i.e., position and orientation). The fold line demarcates the part of the cloth that should be folded from the part that should remain stationary. The goal is then to fold the cloth on the same side as the gripper start pose on top of the cloth on the opposite side.

**Figure 3:**
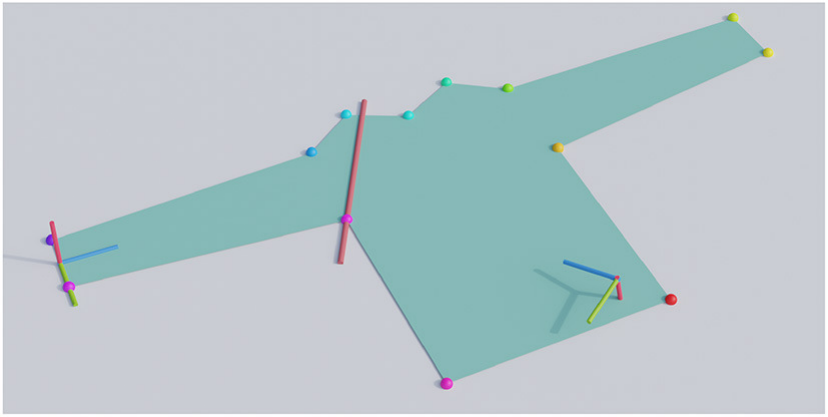
An example fold specification for a sleeve fold on a long-sleeved shirt. The colored dots on the contour of the shirt denote the semantic keypoints. The red line shows the desired fold line. The RGB-colored axes on the left represent the gripper start pose, and those at the right the gripper end pose. The blue axis denotes the forward direction of the gripper.

As mentioned, the gripper start pose and fold line are given relative to a set of semantic 3D keypoints. Various methods are available to detect these keypoints. In settings with simple backgrounds, the contour of the cloth can be easily extracted with traditional techniques and the keypoints can be found by fitting a template to that contour (Miller et al., 2011). More recently deep neural networks have also shown success in cloth keypoint detection (Lips et al., 2022). Figure 3 shows an example fold specification relative to detected keypoints, our sleeve folds start in the center between two keypoints at the end of a sleeve, and the fold line passes through a keypoint in the armpit. All the fold specifications in this work were statically scripted, but we note that it could be interesting to let these be the output of a higher-level decision algorithm.

The gripper end pose is calculated by rotating the start pose 175 degrees around the fold line and then setting its height at 5 cm. We end the fold in this pose slightly above the table to make it easy for the robot to retract its gripper without colliding. We assume that a grasping strategy is used where the gripper approaches from the side and slides underneath the cloth, as in e.g., Jia et al., 2018. We chose this kind of grasp because it is less intrusive and usually causes less localized wrinkling than a top-down pinch. Additionally, note that we do not simulate the grasping of the garment. Instead, we represent the gripper as cuboid that encompasses 2.5 cm of the cloth at the cloth's edge. All triangles that have vertices in this cuboid are considered grasped and move together with the gripper while it executes its trajectory.

We note that the gripper start pose must not necessarily be part of the fold specification, and it could be interesting to algorithmically select an appropriate start pose. However, for the folds we considered in this work, we found it straightforward to script the gripper start poses given the keypoints. Similarly, optimizing the gripper end pose could result in a slight gain in performance.

### 3.2. Trajectory parameterization and search space

The basis of all trajectories in this work is quadratic Bézier curves. Bézier curves are smooth, polynomial interpolations of a set of control points. A Bézier curve of degree *n* has *n*+1 such control points, a quadratic Bézier curve thus has three. A convenient property of Bézier curves is that they pass through the start and end control points. In our case, these are given by the gripper's start and end location. The shape of the curve is then only determined by the remaining middle control point.

Bézier curves are simple and provide us with just the right amount of adaptability. The gravity folds procedure uses a trajectory that consists of two straight parts (Berg et al., 2010). Petrík et al. (2015) proposes a circular trajectory. However, we argue that these fixed trajectories don't have sufficient adaptability (Petrík and Kyrki, 2019). A circular arc has a steep start and keeps the cloth tensioned, this constant tension can be undesirable at times as it can cause severe shifting. In this work, we sought a simple and illustrative trajectory that nevertheless could be effective. Bézier curves were also used for cloth folding by Li et al. (2015). However, they used cubic Bézier curves and thus had two free control points. They optimize the cartesian coordinates of these points and thus have to optimize six degrees of freedom. We decided to reduce the dimensionality of the search space even further, to only two. We do this by constraining the location of this middle control point to lie in the vertical plane that passes through the center between the start and end locations. Note that the fold line also lies in this plane. Moving the middle control point closer to the start or end could produce interesting trajectories, however, for this work we deemed this degree of freedom was not necessary.

**Figure 4:**
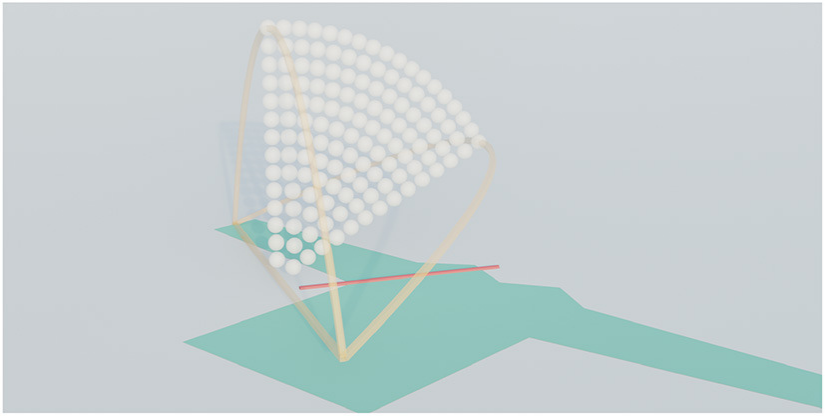
Visualization of the trajectory search space. The white dots indicate the peaks of the trajectories.

The trajectory thus has two remaining degrees of freedom, the height and the horizontal position of the middle control point. Within these two degrees of freedom, we evaluate a set of trajectories from a fan-shaped region, as seen in Figure 4. The fan covers the region we considered where reasonable possibilities for good folding would lie. We considered several possible shapes for the search space but ultimately decided on this fan-shaped region. Each point in this region can be described with two easily interpretable parameters, a height ratio and a tilt angle, similar to how points are specified in polar coordinates. The height ratio determines the height of the peak of the trajectory as a fraction of half the distance from start to end. The tilt angle determines how much the trajectory is rotated around the line that connects the start and end.

The height ratio for the search space goes from 0.1 up to 1.0, the tilt angle goes from 0 up to 60 degrees. There is a trade-off between search space size and how dense it can be sampled, and for this reason, we chose to tilt the trajectories only in one direction. This results in an asymmetric search space. Choosing a direction to tilt proved uncomplicated for all considered folds. For example, for the sleeve folds, the search space is tilted toward the shoulder, as trajectories that tilt away from it would likely cause the shirt to shift.

The motion of the gripper along each trajectory takes exactly 4 s to complete for all scenarios except for three speed variations. We chose this as default because it is comparable to the speed of a person that is folding carefully. The speed of the gripper along the trajectory is also not constant. To ensure a slow and smooth start and end, we use the timing of the minimum jerk trajectory along the path given by the Bézier curve. The longest trajectories are those with height ratio 1. The peak velocity of the gripper along these trajectories in the 4-s case is 0.65 m per second. The orientation of the gripper along the path is determined by spherical linear interpolation (Shoemake, 1985) of the start and end orientation.

### 3.3. Evaluation of the folding trajectories

**Figure 5:**
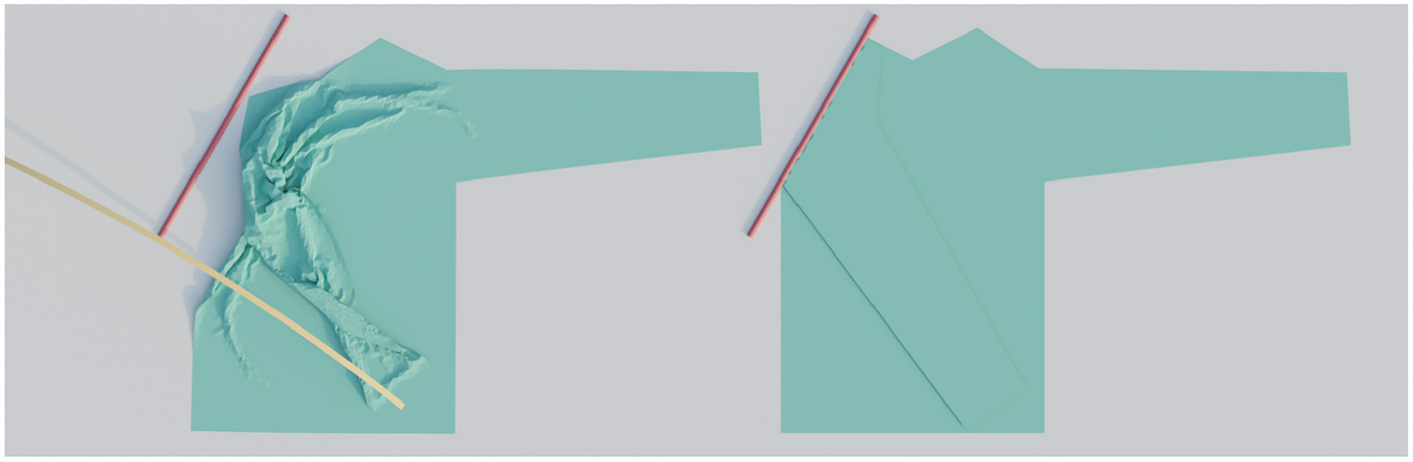
On the left is an example of a simulated result and on the right the idealized target shape. The image on the left has a loss of 2.1 cm, which means the vertices are on average 2.1 cm removed from their desired location.

As the final step of a simulation run, we evaluate the performance of the attempted folding trajectory. We do this by comparing the simulated cloth mesh with an idealized perfectly folded target mesh, shown side-by-side in Figure 5. This target mesh is constructed by geometrically rotating the part to be folded around the fold line by 180 degrees and then raising it slightly, e.g., by the thickness of the chosen fabric. Then we calculate the average distance between each vertex in the simulated mesh to its corresponding location in the target mesh. We will refer to this as the trajectory's loss:


(1) $$\begin{array}{l} \frac{1}{n}\overset{n}{\underset{i=1}{\sum }}\sqrt{{\left({\text{v}}_{i}^{*}-{\text{v}}_{i}\right)}^{2}} \end{array}$$

Here *n* is the amount of vertices in the cloth mesh, **v**_*i*_ the position of the vertex with index *i* in the simulated mesh and ${\text{v}}_{i}^{*}$ the position of the corresponding vertex in the target mesh. Because the loss represents a deviation from an ideal target, high losses are worse than low losses. A loss of 0 means that the simulated result exactly matches the target. For example, we found that a loss of greater than 2 cm for the sleeve folds in this work generally meant that the fold had failed significantly. Losses of around 5 mm generally indicate that fold is already good, but might have minor defects such as small wrinkles. A loss of 2 mm or less corresponds with almost perfect folds that only show negligible and barely visible and defects. The height from the bottom to the top of all shirts is 63.5 cm. Also note that the mean distance values mentioned above are only valid for most sleeve folding cases. The thick material and the different fold steps require different thresholds. A threshold below which a fold can be considered successful should be decided separately for different scenarios.

We chose this mean distance loss because it is simple and captures the two importing failure modes for folding: shifting and wrinkling of the cloth. However, the loss on its own does not tell us whether the fold failed due to shifting or wrinkling. Research into loss functions for cloth folding which disentangle wrinkling and shifting would be highly useful.

### 3.4. Experimental setup

The main purpose of the experiments is to gain general insight into which factors affect folding success. For this reason, we test the performance of the same trajectory search space, but in several scenario variations. By examining which trajectories succeed and which fail in different scenarios, we can learn how much adaptability is necessary for robust cloth folding. The variations we consider are cloth shape, cloth material, the folding step within a folding sequence and folding velocity. In total, we evaluate 13 such scenarios.

For simplicity, all scenarios are variants of a default scenario. The default scenario consists of a shirt with long straight sleeves that are approximately orthogonal to the body of the shirt. This shape was mimicked from a sewing pattern that was bought from a local tailor. The material for this scenario is the type of cotton measured by Penava et al. (2014). The global coefficient of friction was set to 0.5. This governs the friction between the cloth and the ground plane, and the self-friction of the cloth.

The first kind of variation we will consider is the shape of the shirt. We try two shapes different from the default: one with long sleeves, but angled downwards and one with short sleeves. For the material variations, we initially compared the cotton, wool, and polyester materials from Penava et al. (2014). However, the results for wool and polyester were very similar to cotton so we do not include them. Instead, we added more extreme material variations such as cotton which is 5 times as thin or thick. The default cotton has the following material parameters: its Young's modulus is 0.821 GPa, its Poisson's ratio 0.243, its weight 150.3 g/m^2^ and its thickness 0.318 mm. To investigate the influence of friction we test scenarios with global coefficients of friction of 0.2 and 0.8, instead of the default 0.5.

Further, because we are not solely interested in folding sleeves, but entire garments, we investigate an entire 5-step folding sequence. The sequence consists of first folding in the two sleeves, then the two sides, and to conclude a fold over the middle of the shirt. The folding of the sides and the fold over the middle are performed with two grippers that move simultaneously. The simulation of the second folding stage, starts from the best result of the first stage, and the third stage analogously starts from the best second stage result. For the folding sequence to succeed, each individual stage must succeed. If the sleeve folding fails, the folding of the sides will likely also fail.

Finally, different folding velocities might be desirable, depending on the hardware setup or constraints. For this reason, we tested three speed variations for the default sleeve folding scenario. The default length of time to complete the trajectories is 4 s. The variations we test are 1, 2, and 8 s.

## 4. Results

The experiments are presented in three subsections. In the first we examine the three shape variations. The section thereafter presents the four material variations. Then we consider the three stages of the full folding sequence. Finally, the results for the velocity variations are shown.

All experiments were run on a single machine with a 4-core 3.40 GHz Intel i5-4670K CPU. There are 13 scenarios and for each scenario 134 trajectories were tested, so in total 1,340 simulations were run. The total time it took to run all simulations was approximately 405 h, which is about 14 min on average per simulation.

To complement this section we added a Supplementary Video that shows the best and worst simulations for each scenario.

### 4.1. Shape variations

**Figure 6:**
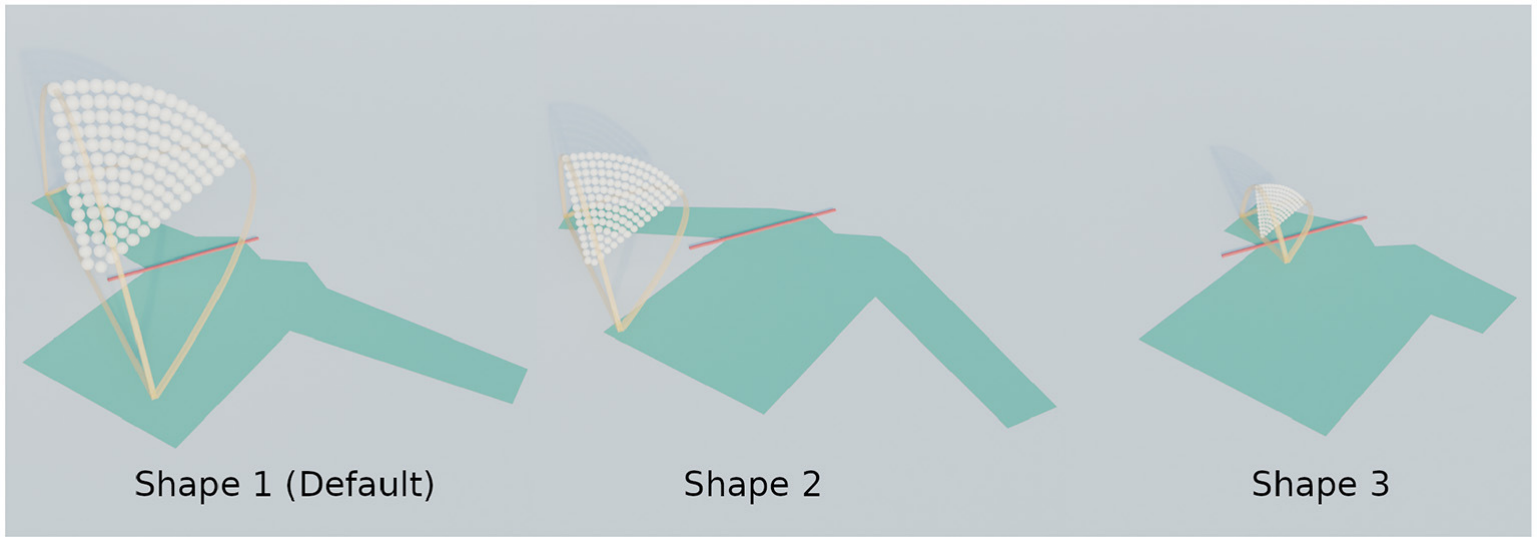
The three examined shapes. Note how the shape of the cloth affects the distance between the start and end points of the fold, and therefore also the spatial size of the search range.

**Figure 7:**
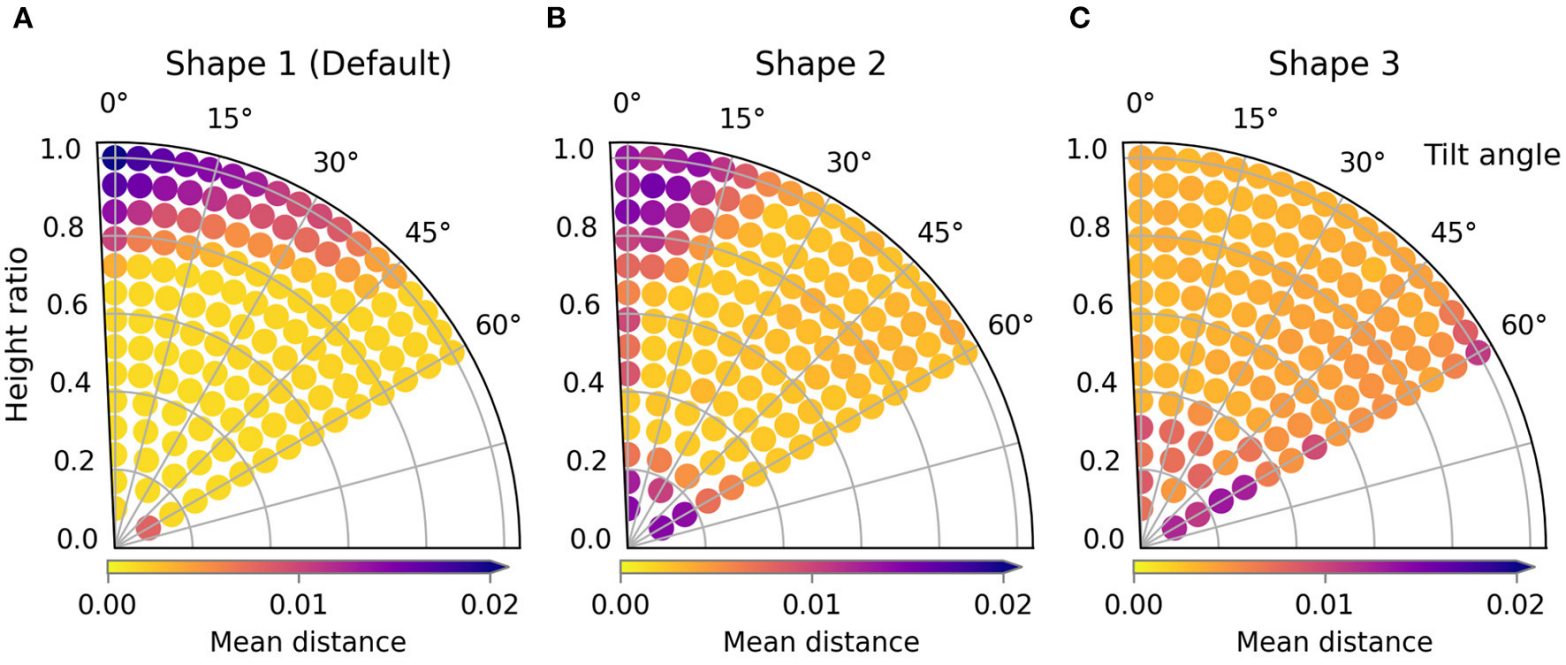
Trajectory performances for the default scenario. The performance for shapes 1, 2, and 3 are shown in **(A)**, **(B)**, and **(C)** respectively.

**Figure 8:**
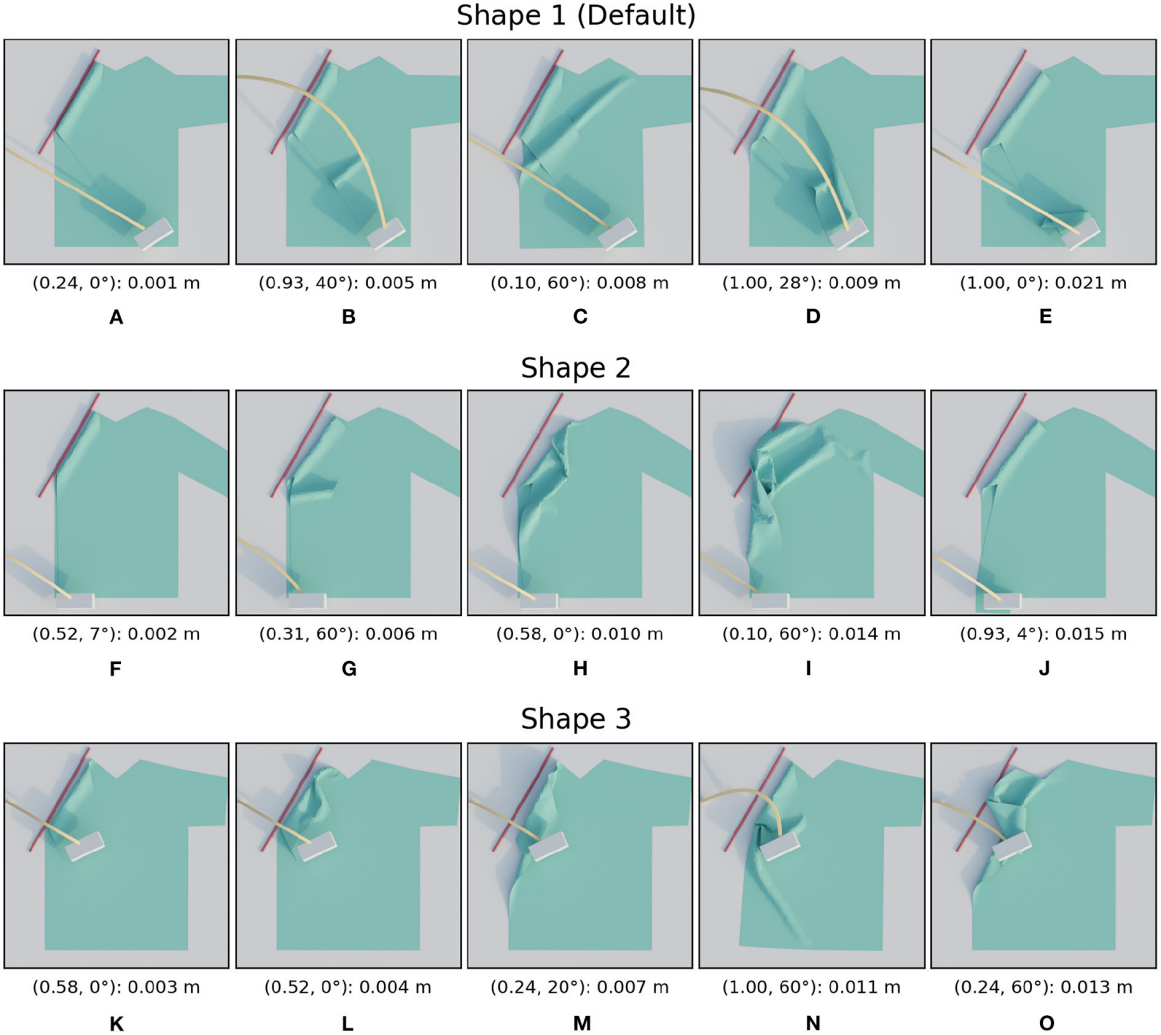
Images of simulated results for the shape variations. For each row, the images are ordered from best (left) to worst (right) result. The annotation below each image is in the following format: (height ratio, tilt angle): mean distance. The first row **(A–E)** shows the images for shape 1, the second row **(F–J)** for shape 2, and the third **(K–O)** for shape 3.

The three shape variations considered in this subsection can be seen in Figure 6. The search space which is derived from each shirt's shape is also visualized. The performance of the trajectories in these search spaces are shown side-by-side in Figure 7 for the shape variations. Additionally, we also show images of several simulation results in Figure 8 as a reference to interpret the loss values and to give insight into the reasons for the failures. The image on the left always corresponds to the best folded result, while the image on the right shows the worst. In between are three manually selected images to show various failures and their loss. Below each image we display the parameter combination and the resulting loss in the following format: (height ratio, tilt angle): mean distance.

When looking at the trajectory performances for shape 1 in Figure 7A we can make several observations. First, we can see that many of the trajectories in the search space perform well. Besides a single trajectory at the bottom, only the trajectories in the top left, those with high height ratio and low tilt result in failed folds. For the worst result we can see that the high loss is because the resulting fold line in the cloth does not coincide with the desired fold line and also that the end of the sleeve is crumpled. The single failure at the bottom is due to a different failure mode: a large wrinkle that appears because the trajectory is so low. This failure can be seen in Figure 8C.

The two shape variations we consider can be seen in Figure 6 as shape 2 and shape 3. The results for these shapes can be seen in Figures 7B,C. For shape 2 we see a similar pattern emerge in the loss landscape as for the default shape, namely the failures mostly occur in the top left and for the lowest trajectories. Also note that for this shape almost no straight trajectories, i.e., those with a tilt angle of 0 degrees, perform well. This means that the extra degree of freedom allowed by tilting is highly beneficial in this case. The results for shape 3 show an entirely different pattern than those of the first two shapes. As opposed to before, the trajectories in the top left do not perform poorly, in fact the third best trajectory is located there, with a height ratio 1 and tilt angle 7 degrees. The lowest trajectories similarly show a few failures. Also notable is that the losses of the bulk of the trajectories for shape 3 are slightly worse than for shape 1 and 2. When looking closely at the image of the best result for shape 3, Figure 8K, a small gap can still be seen between the fold line and the shirt. This is probably due to the end pose of the gripper being slightly suboptimal.

For all tested shape variations we see that there is a large region of well-performing trajectories. Although there are a few failures to be aware of for each shape, there still is overlap between the successful regions. The region in the middle of the search space, from height ratio 0.4 to 0.8 and tilt angle from 15 to 45 degrees, performs well for all shapes. This means that a single static trajectory from that region could fold most sleeves satisfactory for materials that behave similar to the simulated cotton.

### 4.2. Variation of material properties

#### 4.2.1. Thickness variation

**Figure 9:**
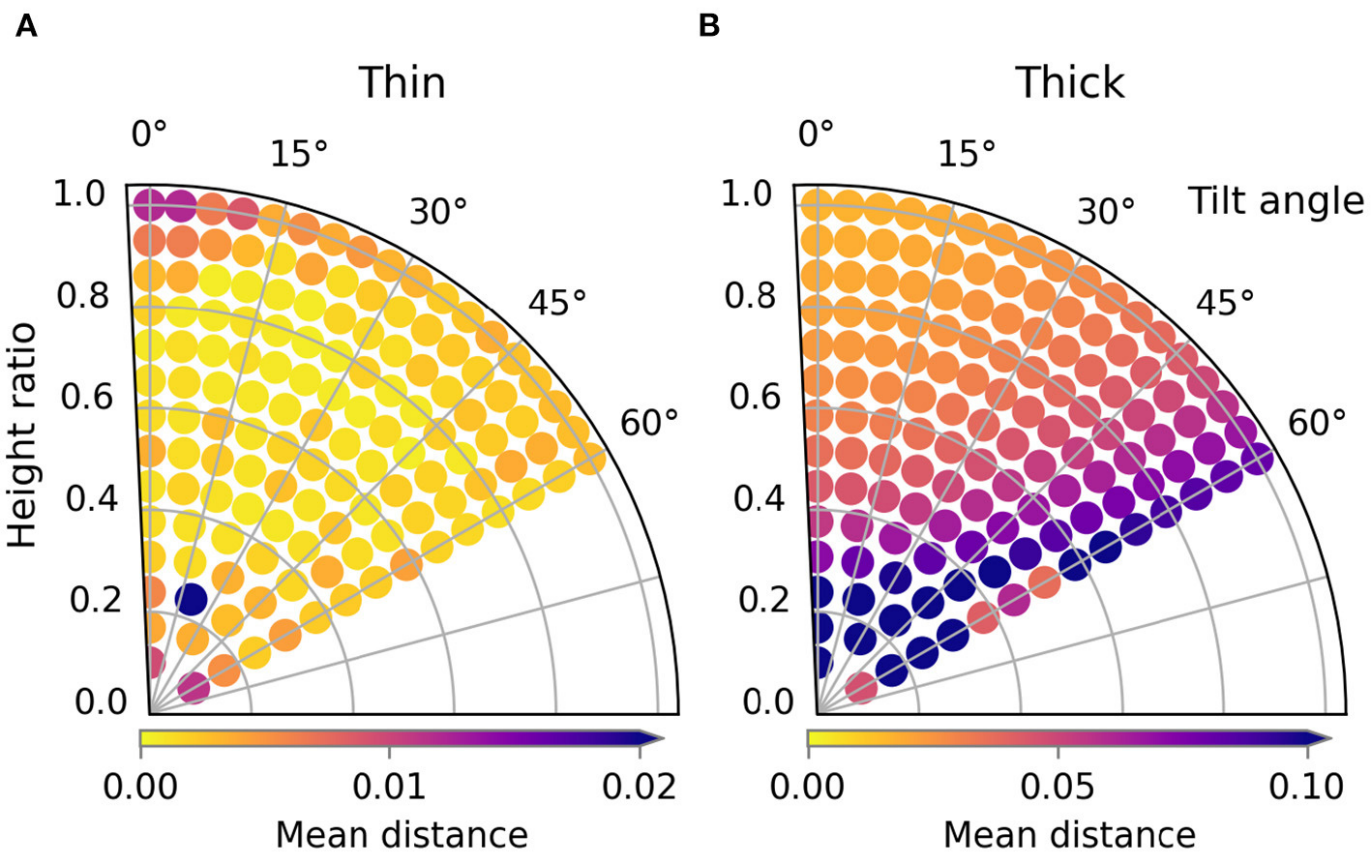
Trajectory performances for the thickness variations. Note that the color scale is different for the thick material compared to the other sleeve folding experiments. The performance for the thin and thick material are shown in **(A)** and **(B)** respectively.

**Figure 10:**
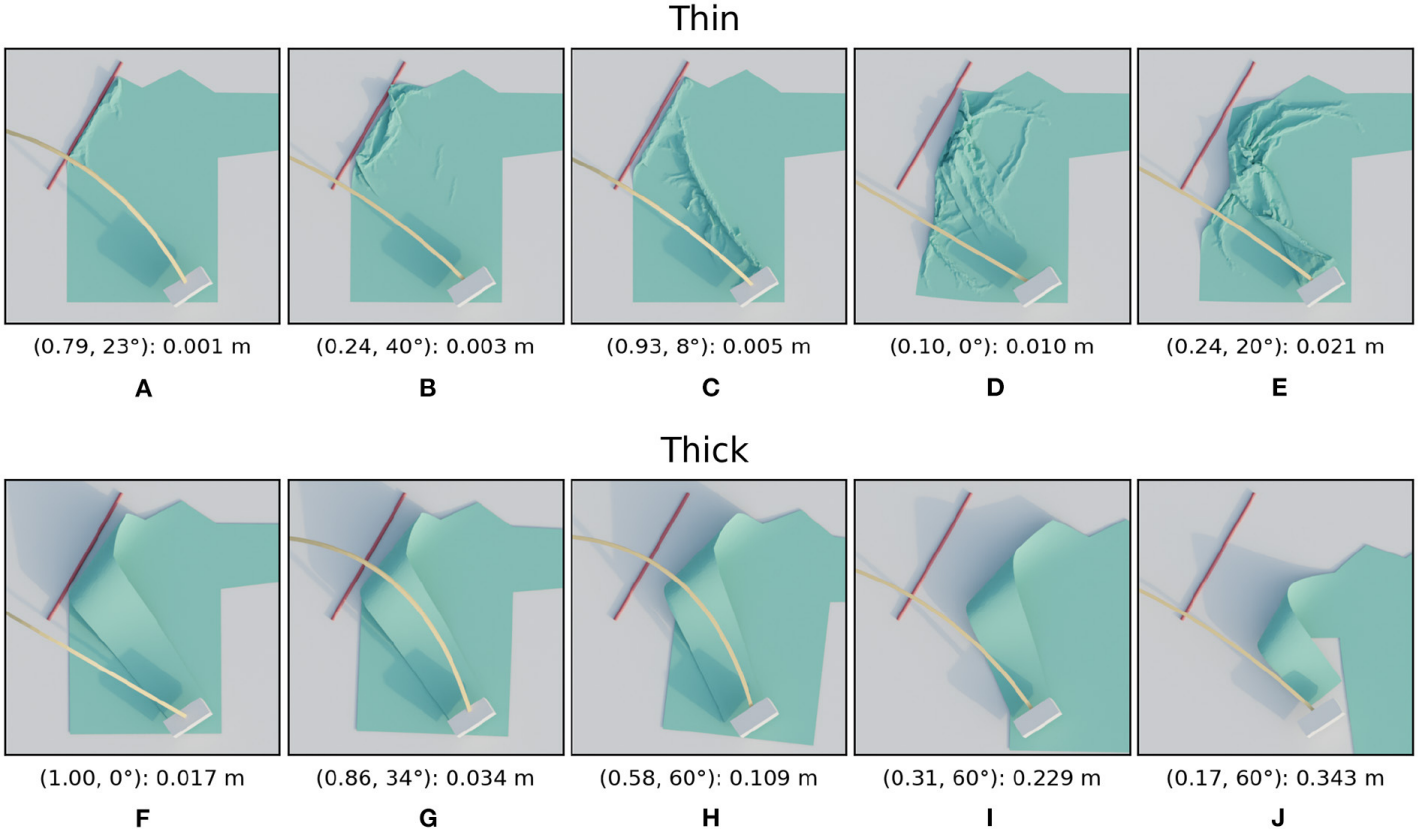
Images of simulated results for the thickness variations. For each row, the images are ordered from best (left) to worst (right) result. The annotation below each image is in the following format: (height ratio, tilt angle): mean distance. The first row **(A–E)** shows the images for the thin material and the second row **(F–J)** for the thick material.

In this subsection, we examine the result of the first type of material variation: the cloth's thickness. Figure 9 shows the trajectory performances for the thin and thick materials, Figure 10 shows images of several simulated results. The loss landscape for the thin material is similar to the that of the default material, except that it is less smooth. This non-smoothness is because the thin material allows much finer wrinkles to form. The occurrence and magnitude of these fine wrinkles appears chaotic, trajectories with higher loss than their neighbors are speckled throughout the loss landscape. The trajectory of the worst result is near the bottom at height ratio 0.24 and tilt angle 20 degrees. This particular trajectory causes the sleeve to twist, which causes a large amount of wrinkling, as can be seen in Figure 10E.

The losses for the thick material were significantly higher for all trajectories. So to ensure the variability of loss landscape remained visible, note that the maximum of the color bar range is five times higher than for the other sleeve folding scenarios. The reason that even the best trajectories have higher losses than those of the thinner shirts is because the thickness of the material causes the sleeve to bend with a large radius of curvature. Visually this reminds of how paper or leather bends. The loss landscape in general shows a unique and clear pattern: the trajectory in the top left performs best and performance gradually drops as you get further away. For an unclear reason a few trajectories with tilt angle 60 degrees seem to break this trend by having lower losses than the surrounding trajectories. As can also be see for the worst result, the failures for the thick material are all due to the shirt sliding. The thickness of the material prevents the formation of wrinkles, which causes the horizontal force of the gripper on the shirt to build up. When this horizontal force surpassed the maximum static friction, the shirt starts to slide.

It is clear that material thickness has a large effect on trajectory performance. This is because cloth thickness strongly influences which failure mode is dominant: thin material tends to wrinkle and thick material tends to slide. It thus seems highly necessary to adapt the folding trajectory based on information about the thickness of the garment to be folded.

#### 4.2.2. Friction variation

**Figure 11:**
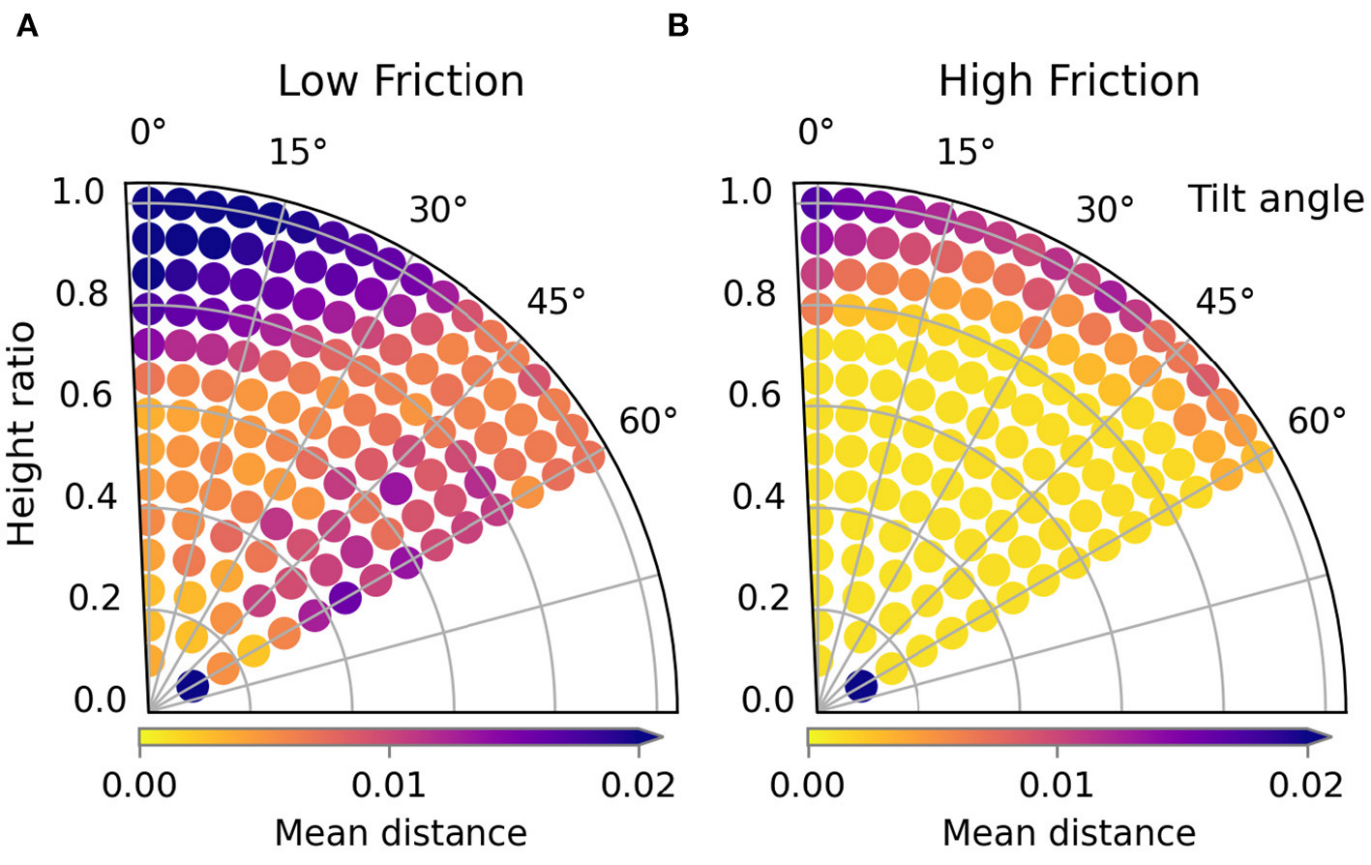
Trajectory performances for the friction variations. The performance for the low friction and high friction scenario are shown in **(A)** and **(B)** respectively.

**Figure 12:**
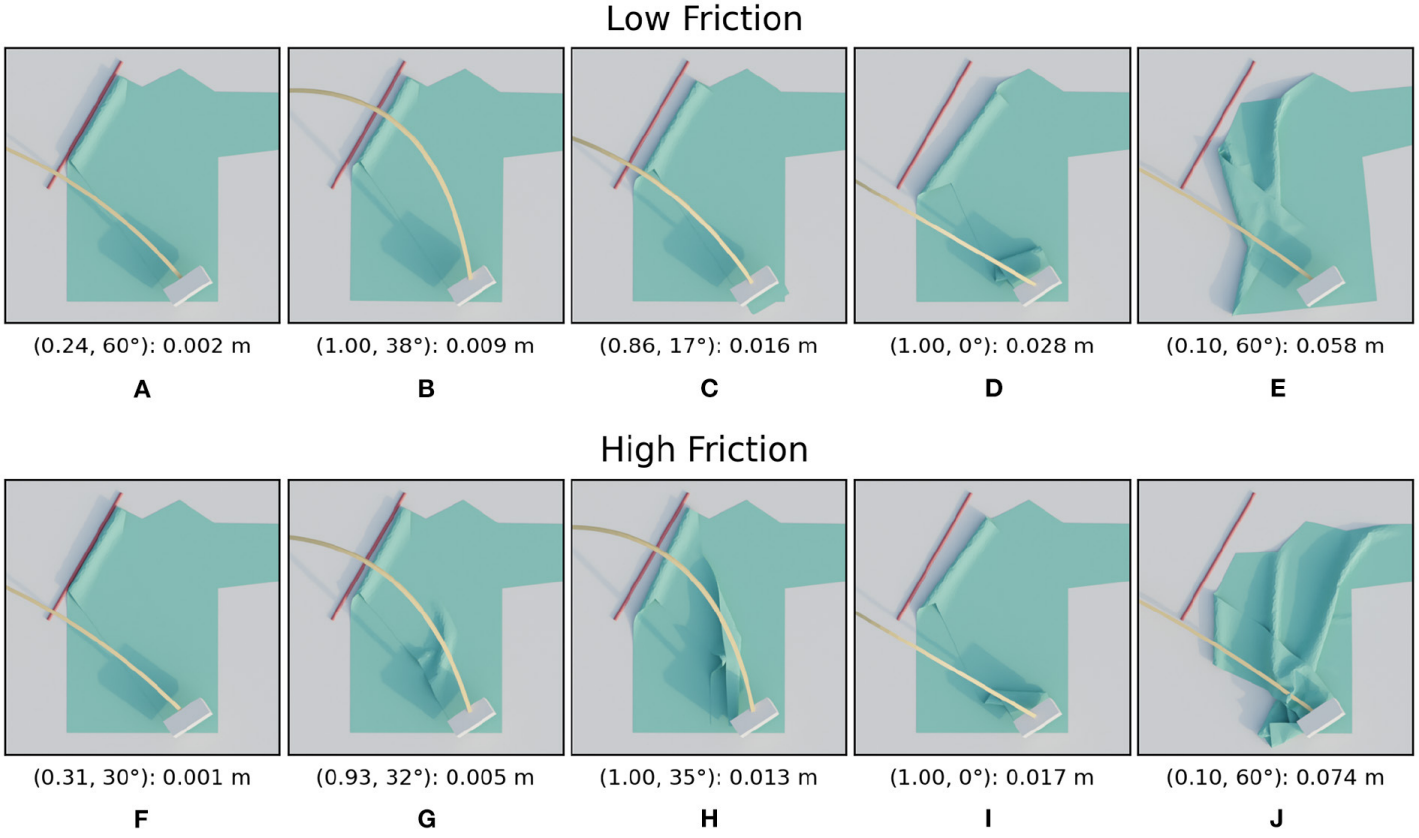
Images of simulated results for the friction variations. For each row, the images are ordered from best (left) to worst (right) result. The annotation below each image is in the following format: (height ratio, tilt angle): mean distance. The first row **(A–E)** shows the images for the low friction scenario and the second row **(F–J)** for the high friction scenario.

The trajectory performances for the second type of material variation, friction, are shown in Figure 11, the images are shown in Figure 12. For the low friction scenario we can see that the loss landscape differs from the previous scenarios. The top left and the middle right are clearly low performance areas. The best region seems to be a small band around height ratio 0.2. The worst trajectory is the lowest one at the bottom and shows severe crumpling. However this is only a single instance, the main failure mode is simply that the sleeve is folded too far which also causes an incorrect fold line.

For the high friction scenario results we can see that the loss landscape has not changed much compared to the default friction. Almost all low and medium high trajectories result in highly successful folds in both scenarios. One difference is that the worst trajectory by far for the high friction scenario is the lowest trajectory.

From the three tested friction variations we can conclude that folding with low friction is significant more complex than folding with medium or high friction.

### 4.3. Folding sequence

**Figure 13:**
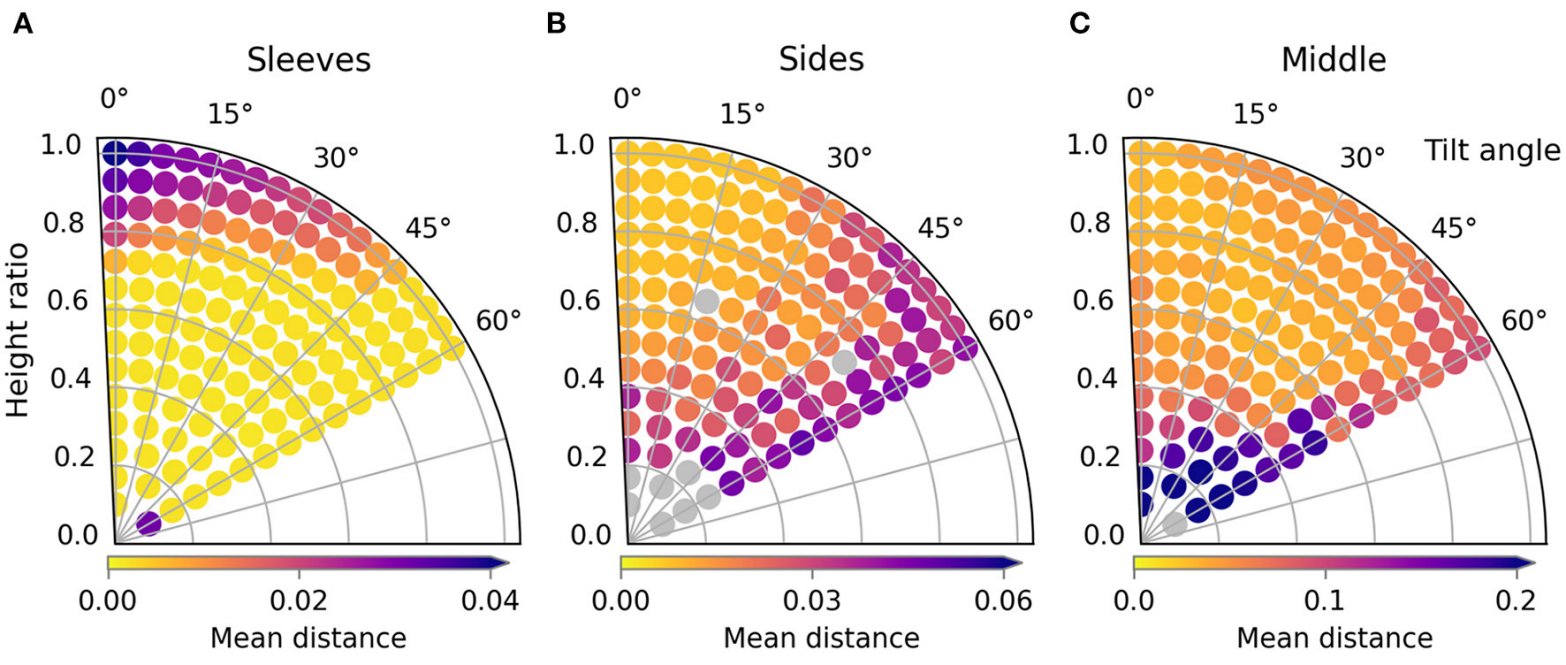
Trajectory performances for the three phases of the folding sequence: the folding of the sleeves **(A)**, the sides **(B)**, and then the final fold over the middle **(C)**. The material used in this sequence is the default cotton. The gray dots in the figures represent simulations for which no loss was calculated because they crashed due to forced self-intersection of the cloth.

**Figure 14:**
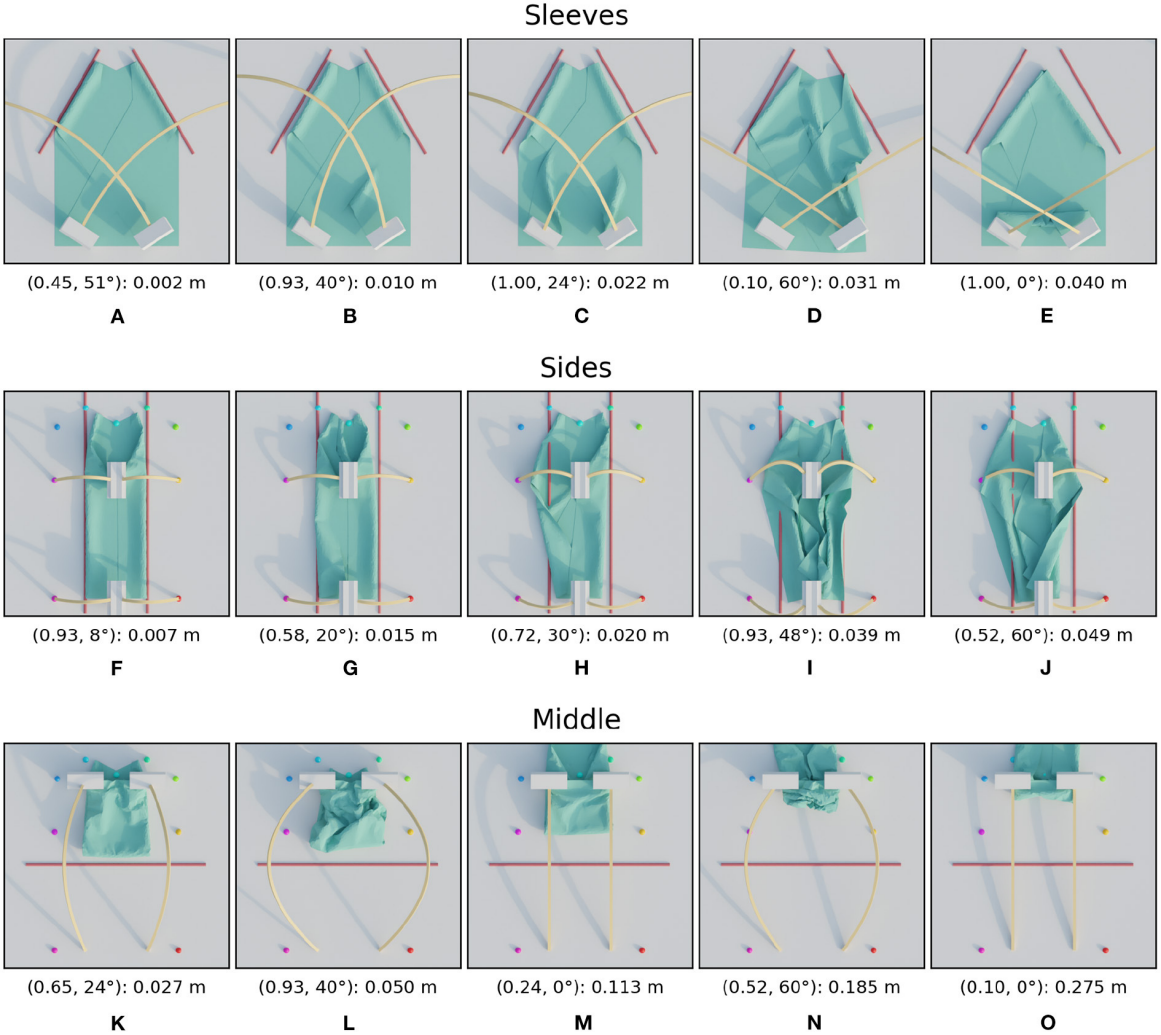
Images of simulated results for the three phases of the folding sequence. For each row, the images are ordered from best (left) to worst (right) result. The annotation below each image is in the following format: (height ratio, tilt angle): mean distance. The first row **(A–E)** shows the images for the sleeves, the second row **(F–J)** for the sides, and the third **(K–O)** for the middle. Note that the side folding stage starts from the cloth as seen in **(A)**, the best result from the sleeve folding. The middle folding similarly starts from **(F)**, the best result from the side folding.

In this subsection, we discuss the results of the three stages of a full folding sequence of the default long-sleeved shirt. The trajectory performances are shown in Figure 13, the images in Figure 14. In the first stage, both sleeves are folded in. We can see that as expected, the loss landscape for folding two sleeves is very similar to the folding a single sleeve.

More interesting are the results for the second stage where the sides of the shirt are folded in. Firstly, note the gray dots in the loss landscape which represent missing data due to failed simulations. Simulations can fail if the gripper forces the part of the cloth it holds to intersect with another part of the cloth. C-IPC prevents these intersections for freely moving cloth parts, but can't recover from forced intersections. The region with trajectories that performs poorly for the sides is completely different than that for the sleeves. For the sleeves, the high, straight trajectories led to the worst folds, while for the sides these give the best results. Tilting the trajectories also seems universally bad for folding the sides.

For the final fold in the sequence, i.e., the fold over the middle of the shirt, we can see that the lowest trajectories all perform poorly. The trajectories tilted close to 60 degrees also show relatively high losses. In addition to those failures, there is a large region in the middle and top left with good performance.

The results for the full folding sequence show that there are trajectories within in the search space that lead to nicely folded results for all folding steps. Furthermore, the loss landscapes show that for each fold in the sequence, a relatively large region of high performance exists. These regions are also distinct and specific for each folding step. This indicates that is beneficial to differentiate the folding strategy between steps.

### 4.4. Velocity variation

**Figure 15:**
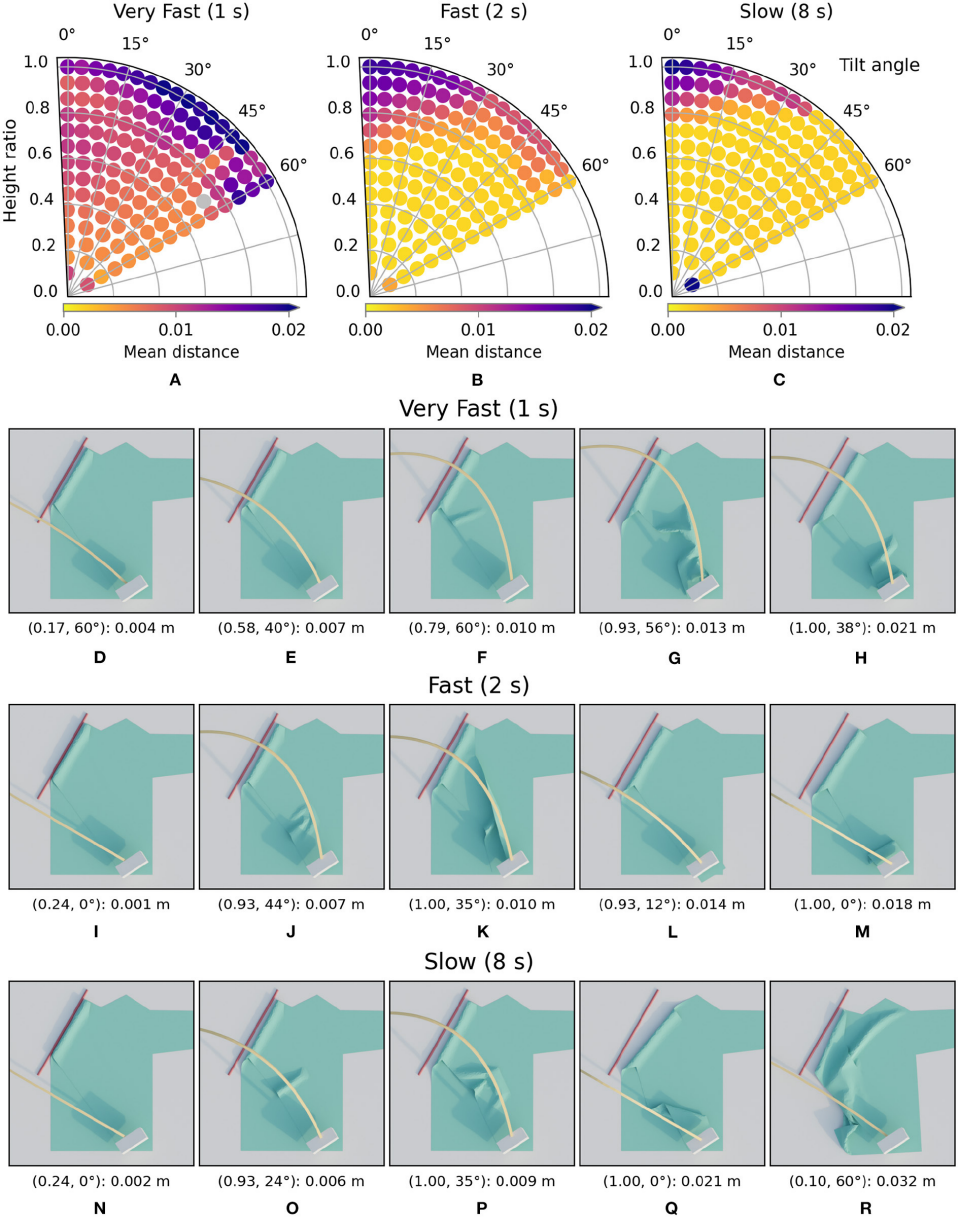
The first row shows the trajectory performances **(A–C)** for the velocity variations respectively 1, 2, and 8 s. Below **(D–R)** are the images of simulated results for the velocity variations. For each row, the images are ordered from best (left) to worst (right) result. The annotation below each image is in the following format: (height ratio, tilt angle): mean distance.

The effect of folding velocity on performance and the corresponding images can be seen in Figure 15. In the performance plots we can see that when folding slower, with 8-s trajectories, the region with well-performing trajectories is largest. When the velocity is increased, such that the trajectory takes 2 s, we can see that many trajectories still achieve low loss values. However, the region with failures is already larger than in default 4-s case. When increasing velocity even further such that the folds only take 1 s, the loss landscape changes significantly. The region with good performance in the 2, 4, and 8 s cases has almost entirely disappeared. In this setting, the gripper moves so fast that the cloth almost always moves too far due to its inertia. For very high speed cloth folding, different paths for the trajectory might yield better results. At moderate speeds the current trajectories can achieve many successful folds.

## 5. Discussion

In this work, we thoroughly investigated cloth folding in the high fidelity C-IPC simulator. We found that with a relatively simple trajectory-based approach, we can successfully perform a full cloth folding sequence. We were able to characterize the folding trajectories with two parameters: one parameter for the trajectory's height and one parameter to tilt it. For all scenarios we investigated, we found that there are trajectories in our search space that lead to a satisfactory folded result. However, we also found that adapting the trajectory to the cloth shape, cloth material, and the folding step is crucial for success. Consequently, for a transfer to a real-world scenario, it will be important to estimate the cloth's properties so that the parameters can be tuned correctly. Several solutions can be tried, such as material estimation from video or other sensors, or closed-loop controllers that can adapt to unpredicted motions of the cloth. However, even with fitted parameters, discrepancies between simulation and reality are unavoidable.

To use our method for practical real world folding, an additional system is needed that selects a suitable trajectory from observations. As a first step, one could simply select a single high-performing trajectory from a default simulated shirt, and use it to fold a real-world shirt. More advanced systems could perceive more information about the present garment. The shape could be estimated using keypoint detection and the material properties could be approximated from tactile information of short interactions. With this information the system could then look for an appropriate trajectory, e.g., through nearest neighbor search. It is thus not necessary to run the computationally expensive simulator at runtime to use our method.

To use our method for practical real world folding, an additional system is needed that selects a suitable trajectory from observations. As a first step, one could simply select a single high-performing trajectory from a default simulated shirt, and use it to fold a real-world shirt. More advanced systems could try to estimate the cloth shape and approximate material properties, e.g., from cameras, tactile information and short interactions. Then the system could use a lookup table or nearest neighbor search in a dataset of precomputed simulations to select an appropriate trajectory. It is thus not necessary to run the computationally expensive simulator at runtime to use our method.

In future work, we will investigate the robustness of trajectories transferred from simulation to real-world setups. We believe that by using simulation that is validated against real-world behavior we can, in time, give robots the capability to autonomously and reliably fold clothes.

## Data availability statement

The original contributions presented in the study are included in the article/Supplementary material, further inquiries can be directed to the corresponding author/s.

## Author contributions

V-LD and FW contributed to conception and design of the experiments. V-LD implemented and ran the experiments and wrote the first draft of the manuscript. Both authors contributed to manuscript revision, read, and approved the submitted version.

## Funding

This research was partially funded by the Research Foundation Flanders (FWO) under Grant Number 1SD4421N and the AI for Flanders program.

## Conflict of interest

The authors declare that the research was conducted in the absence of any commercial or financial relationships that could be construed as a potential conflict of interest.

## Publisher's note

All claims expressed in this article are solely those of the authors and do not necessarily represent those of their affiliated organizations, or those of the publisher, the editors and the reviewers. Any product that may be evaluated in this article, or claim that may be made by its manufacturer, is not guaranteed or endorsed by the publisher.

## References

[B1] Antonova R. Shi P. Yin H. Weng Z. Jensfelt D. K. (2021). “Dynamic environments with deformable objects,” in Thirty-fifth Conference on Neural Information Processing Systems Datasets and Benchmarks Track (Round 2), eds J. Vanschoren, and S. Yeung. Available online at: https://datasets-benchmarks-proceedings.neurips.cc/paper/2021/hash/b53b3a3d6ab90ce0268229151c9bde11-Abstract-round2.html

[B2] Berg J. Miller S. Goldberg K. Abbeel P. (2010). “Gravity-based robotic cloth folding,” in Algorithmic Foundations of Robotics IX, eds D. Hsu, V. Isler, J.-C. Latombe, and M. C. Lin (Berlin; Heidelberg: Springer), 409–424. 10.1007/978-3-642-17452-0_24

[B3] Doumanoglou A. Stria J. Peleka G. Mariolis I. Petrik V. Kargakos A.. (2016). Folding clothes autonomously: a complete pipeline. IEEE Trans. Robot. 32, 1461–1478. 10.1109/TRO.2016.2602376

[B4] Henderson P. Islam R. Bachman P. Pineau J. Precup D. Meger D. (2018). “Deep reinforcement learning that matters,” in Proceedings of the AAAI Conference on Artificial Intelligence (New Orleans). 10.1609/aaai.v32i1.11694

[B5] Jangir R. Alenya G. Torras C. (2020). “Dynamic cloth manipulation with deep reinforcement learning,” in 2020 IEEE International Conference on Robotics and Automation (ICRA) (Paris: IEEE), 4630–4636. 10.1109/ICRA40945.2020.9196659

[B6] Jia B. Hu Z. Pan J. Manocha D. (2018). “Manipulating highly deformable materials using a visual feedback dictionary,” in 2018 IEEE International Conference on Robotics and Automation (ICRA) (Brisbane, QLD: IEEE), 239–246. 10.1109/ICRA.2018.8461264

[B7] Jiménez P. (2017). Visual grasp point localization, classification and state recognition in robotic manipulation of cloth: an overview. Robot. Auton. Syst. 92, 107–125. 10.1016/j.robot.2017.03.009

[B8] Kadian A. Truong J. Gokaslan A. Clegg A. Wijmans E. Lee S.. (2020). Sim2real predictivity: does evaluation in simulation predict real-world performance? IEEE Robot. Automat. Lett. 5, 6670–6677. 10.1109/LRA.2020.3013848

[B9] Kim C. M. Danielczuk M. Huang I. Goldberg K. (2022). “Simulation of parallel-jaw grasping using incremental potential contact models,” in 2022 IEEE International Conference on Robotics and Automation (Philadelphia, PA). 10.1109/ICRA46639.2022.9811777

[B10] Li M. Ferguson Z. Schneider T. Langlois T. R. Zorin D. Panozzo D.. (2020). Incremental potential contact: intersection-and inversion-free, large-deformation dynamics. ACM Trans. Graph. 39, 49. 10.1145/3386569.3392425

[B11] Li M. Kaufman D. M. Jiang C. (2021). Codimensional incremental potential contact. ACM Trans. Graph. 40, 1–24. 10.1145/3476576.3476756

[B12] Li Y. Yue Y. Xu D. Grinspun E. Allen P. K. (2015). “Folding deformable objects using predictive simulation and trajectory optimization,” in 2015 IEEE/RSJ International Conference on Intelligent Robots and Systems (IROS) (Hamburg: IEEE), 6000–6006. 10.1109/IROS.2015.7354231

[B13] Lin X. Wang Y. Olkin J. Held D. (2021). “Softgym: benchmarking deep reinforcement learning for deformable object manipulation,” in Proceedings of the 2020 Conference on Robot Learning, eds J. Kober, F. Ramos, and C. Tomlin (PMLR). Available online at: https://proceedings.mlr.press/v155/

[B14] Lips T. De Gusseme V.-L. Wyffels F. (2022). “Learning keypoints from synthetic data for robotic cloth folding,” in 2022 International Conference on Robotics and Automation (ICRA) (Philadelphia), 4.

[B15] Maitin-Shepard J. Cusumano-Towner M. Lei J. Abbeel P. (2010). “Cloth grasp point detection based on multiple-view geometric cues with application to robotic towel folding,” in 2010 IEEE International Conference on Robotics and Automation (IEEE), 2308–2315. 10.1109/ROBOT.2010.5509439

[B16] Matas J. James S. Davison A. J. (2018). “Sim-to-real reinforcement learning for deformable object manipulation,” in Conference on Robot Learning (Zürich: PMLR), 734-743.

[B17] Miller S. Fritz M. Darrell T. Abbeel P. (2011). “Parametrized shape models for clothing,” in 2011 IEEE International Conference on Robotics and Automation (Shanghai: IEEE), 4861–4868. 10.1109/ICRA.2011.5980453

[B18] Narain R. Samii A. O'brien J. F. (2012). Adaptive anisotropic remeshing for cloth simulation. ACM Trans. Graph. 31, 1–10. 10.1145/2366145.2366171

[B19] Penava Ž. Šimić-Penava D. Knezic Ž. (2014). Determination of the Elastic Constants of Plain Woven Fabrics by a Tensile Test in Various Directions. Fibres & Textiles in Eastern Europe.

[B20] Petrík V. Kyrki V. (2019). “Feedback-based fabric strip folding,” in 2019 IEEE/RSJ International Conference on Intelligent Robots and Systems (IROS) (Macau: IEEE), 773–778.

[B21] Petrík V. Smutnỳ V. Krsek P. Hlaváč V. (2015). “Robotic garment folding: precision improvement and workspace enlargement,” in Conference Towards Autonomous Robotic Systems (Liverpool: Springer), 204–215. 10.1007/978-3-319-22416-9_25

[B22] Petrík V. Smutnỳ V. Krsek P. Hlaváč V. (2017). Single arm robotic garment folding path generation. Adv. Robot. 31, 1325–1337. 10.1080/01691864.2017.1367325

[B23] Seita D. Florence P. Tompson J. Coumans E. Sindhwani V. Goldberg K.. (2021). “Learning to rearrange deformable cables, fabrics, and bags with goal-conditioned transporter networks,” in 2021 IEEE International Conference on Robotics and Automation (ICRA) (IEEE), 4568–4575. 10.1109/ICRA48506.2021.9561391

[B24] Shoemake K. (1985). “Animating rotation with quaternion curves,” in Proceedings of the 12th Annual Conference on Computer Graphics and Interactive Techniques (San Francisco, CA), 245–254. 10.1145/325334.325242

[B25] Stria J. Prusa D. Hlavac V. Wagner L. Petrik V. Krsek P.. (2014). “Garment perception and its folding using a dual-arm robot,” in 2014 IEEE/RSJ International Conference on Intelligent Robots and Systems (Chicago, IL), 61–67. 10.1109/IROS.2014.6942541

[B26] Verleysen A. Biondina M. Wyffels F. (2022). Learning self-supervised task progression metrics: a case of cloth folding. Appl. Intell. 10.1007/s10489-022-03466-8. Available online at: https://link.springer.com/article/10.1007/s10489-022-03466-8#citeas

